# The Role of *Chlamydia trachomatis* Polymorphic Membrane Proteins in Inflammation and Sequelae among Women with Pelvic Inflammatory Disease

**DOI:** 10.1155/2011/989762

**Published:** 2011-10-19

**Authors:** Brandie D. Taylor, Toni Darville, Chun Tan, Patrik M. Bavoil, Roberta B. Ness, Catherine L. Haggerty

**Affiliations:** ^1^Department of Epidemiology, Graduate School of Public Health, University of Pittsburgh, Pittsburgh, PA 15261, USA; ^2^Department of Epidemiology, Michigan State University, 644 West Fee Hall, East Lansing, MI 48824, USA; ^3^Department of Pediatrics, University of Pittsburgh Medical Center, Pittsburgh, PA 15224, USA; ^4^Department of Microbial Pathogenesis, University of Maryland Dental School, Baltimore, MD 21201, USA; ^5^School of Public Health, The University of Texas Health Science Center, Houston, TX 77030, USA

## Abstract

*Chlamydia trachomatis* polymorphic membrane proteins (Pmps) may increase genital tract inflammation and play a role in virulence. Antibody levels for PmpA, PmpD, and PmpI, measured in densitometric units, were assessed among a pilot sample of 40 *C. trachomatis*-infected women with mild-to-moderate clinical PID. Women who expressed antibodies to PmpA were less likely to achieve pregnancy (40.0% versus 85.7%; *P* = 0.042) and less likely to have a live birth (0.0% versus 80.0%; *P* = 0.005) compared to women who did not express antibody to PmpA. Women who expressed antibodies to PmpI were more likely to have upper genital tract infection (61.5% versus 20.0%; *P* = 0.026). However, seropositivity to PmpI and PmpD did not modify the risk of reproductive sequelae or inflammation. Seropositivity to chlamydial PmpA may represent a biomarker of increased risk of sequelae secondary to infection with *C. trachomatis*.

## 1. Introduction

*Chlamydia trachomatis* is the most common bacterial sexually transmitted infection in the United States [1]. In women, *C. trachomatis* can ascend from the endocervix to the upper genital tract and cause pelvic inflammatory disease (PID) and serious reproductive morbidity including infertility and ectopic pregnancy [2]. However, rates of progression vary and 80% or more of women with chlamydia do not develop PID [1]. Some women clear chlamydial infection without tissue damage, while in some cases *C. trachomatis* induces a chronic low-grade infection [3]. This may lead to persistent inflammation of the upper genital tract causing long-term reproductive sequelae. The pathogenesis of *C. trachomatis *disease is not well-understood, and pathogen-specific virulence factors that may contribute to variability in the course and outcome of infection have not been identified. 

Nine surface-exposed *C. trachomatis* polymorphic membrane proteins (Pmps) are encoded via a multigene family yielding PmpA to PmpI [4]. Pmps represent 13.6% of the coding capacity of the *C. trachomatis* genome [4], suggesting they have a critical role in biology and virulence [5, 6]. However, the role of Pmps in chlamydial virulence is not well understood. PmpD is a species-common, pan-neutralizing antigen hypothesized to hold potential as a vaccine candidate [6]. Thus, the development of high titers of antibody to PmpD might protect from infection or disease. On the other hand, *Chlamydia pneumoniae *Pmps have been shown to induce proinflammatory mediators in infected host cells, demonstrating the potential for these proteins to play a direct role in pathogenesis [7, 8]. All nine Pmps are expressed on the surface of chlamydial elementary bodies (EB) and *C. trachomatis*-infected patients can produce antibodies to each Pmp subtype [9]. However, antibody profiles vary among *C. trachomatis*-infected patients [5]. In addition, comparative genomics has revealed genetic variation and rearrangements among *pmp *gene families in different strains and isolates [5, 9]. This suggests that immune pressure leads to antigenic variation in these surface-exposed proteins [5, 9], a further indication that these proteins have a role in chlamydial virulence. 

Pmps may be involved in virulence, but very little is known about their role in the development of PID and adverse reproductive sequelae. Tan et al. examined variation in Pmp-specific antibody responses in four distinct patient populations, demonstrating that women with PID had significantly higher reactivity to PmpB and PmpI compared to adolescent females with lower genital tract infection [5]. These data may reflect a role for these specific Pmps in inflammation, or simply that women with PID had sustained increased exposure due to repeated or chronic infection. 

In a separate study, Tan et al. found that Pmps exhibit on/off switching *in vitro* which enables independent expression of each Pmp [9]. PmpA, PmpD, and PmpI had very low “off” frequencies of 0.5–1%, suggesting that expression of these Pmps provides an *in vitro* phenotypic advantage [9]. This may or may not translate into enhanced *in vivo* virulence. The high “on” frequencies of PmpD and PmpI correlate with the fact that anti-PmpD and -PmpI antibodies are commonly detected in *C. trachomatis-*infected patients. However, despite the high “on” frequency of PmpA, anti-PmpA antibodies are relatively rare. Further research from this group found that Pmp transcriptional units are differentially expressed during chlamydial development [10]. In addition, Pmp expression was altered under penicillin-induced stress, except for the expression of PmpA, PmpD, and PmpI, which remained steady [10]. Taken together, these data suggest an importance for PmpA, PmpD, and PmpI in chlamydial pathogenesis. However, the role of these Pmps in chlamydial PID has never been examined in humans. In order to examine the role of PmpA, PmpD, and PmpI in chlamydial pathogenesis, we conducted a pilot study to determine if antibody responses specific for these Pmps were associated with parameters of inflammation or sequelae in a group of women with documented *C. trachomatis *PID. 

## 2. Methods

This study utilized data from the PID evaluation and clinical health (PEACH) study. This was the first randomized clinical trial to compare inpatient and outpatient treatment in preventing long-term complications among 831 women with mild-to-moderate PID. The methods of subject recruitment, data collection, and followup have been reported elsewhere [11]. Briefly, between March 1996 and February 1999, women aged 14–37 years were recruited from emergency departments, obstetrics and gynecology clinics, sexually transmitted disease clinics, and private practices at 7 primary and 6 secondary sites throughout the eastern, southern, and central regions of the United States. Women who had suspected PID and gave informed consent were eligible for the PEACH study. Women were enrolled on the basis of clinically generalizable criteria for suspected PID. Eligibility included a history of pelvic discomfort for less than 30 days, findings of pelvic organ tenderness (uterine or adnexal) on bimanual examination, and leukorrhea and/or mucopurulent cervicitis and/or untreated but documented gonococcal or chlamydial cervicitis. The University of Pittsburgh Institutional Review Board approved the study.

A total of 2941 women were screened for study entry, of those 346 (11.8%) did not meet the clinical inclusion criteria for randomization. Women were additionally excluded if they were pregnant (*n* = 141, 4.8%); had taken antimicrobials within the past 7 days (*n* = 248, 8.4%); had a history of hysterectomy or bilateral salpingectomy (*n* = 248, 8.4%); had an abortion, delivery, or gynecologic surgery within the past 14 days (*n* = 51, 1.7%); had a suspected tubo-ovarian abscess or other condition requiring surgery (*n* = 191, 6.5%); had an allergy to the study medications (*n* = 163, 5.5%); were homeless (*n* = 29, 1%); or had vomiting after a trial of antiemetic treatment (*n* = 11, 0.4%). A total of 831 were enrolled and were contacted at least once after randomization. Our analysis included a pilot sample of 40 *C. trachomatis*-positive women, whose serum samples were previously analyzed for Pmp antibodies [5]. All sera were collected at baseline. 

Women were randomized to either inpatient treatment of intravenous cefoxitin every 6 hours and doxycycline orally twice a day for 14 days; or outpatient treatment consisting of a single intramuscular injection of cefoxitin and oral doxycycline twice a day for 14 days. Because the treatment modality was not associated with reproductive morbidities in the PEACH study [12], we do not include them as a covariate in this analysis. Participants were followed-up with in-person visits at 5 and 30 days after treatment. At the 30-day followup, the gynecological exam was repeated. Telephone followups were conducted by the study nurses every 3 months during the first year after enrollment and then every 4 months until June 2004. At that point, information was obtained by self-report for 69.1% of the cohort, with a mean followup of 84 months. 

A pelvic examination and interview were conducted at the baseline visit. The interview collected information on reason for visit, brief pain history, demographics, history of PID/sexually transmitted diseases, sexual and contraceptive history, reproductive decisions, douching history, pregnancy history, medical and gynecological history, and lifestyle habits. Followup interviews collected self-reported information on pelvic pain, pregnancy and births, signs and symptoms of PID, STDs, contraceptive use, pattern of sexual intercourse, and health care utilization. 

Gynecological examinations were performed at baseline and 5 and 30 days after treatment. Endometrial biopsy and cervical swab specimens were obtained for histological examination including chlamydial polymerase chain reaction (PCR) and gonococcal culture. All cultures and PCR were performed at a central reference laboratory. For the patients with endometrial biopsies, two reference pathologists separately evaluated at least one section stained with hematoxylin and eosin and at least one stained with methyl green pyronin. A disagreement about the presence or absence of neutrophils and plasma cells was settled by both pathologists reading the slides together and coming to an agreement. Histological endometritis was based on a modification of the criteria proposed by Kiviat et al. [13]. Endometritis was defined as the presence of at least five neutrophils in the endometrial surface epithelium in the absence of menstrual endometrium and/or at least two plasma cells in the endometrial stroma. This definition has been found to be the best predictor of upper genital tract infection plus salpingitis, with a sensitivity of 92% and specificity of 87% [13]. 

Reproductive outcomes were assessed over a mean of 84 months. Measures of fertility included infertility, live birth, and pregnancy. Other reproductive outcomes included recurrent PID and chronic pelvic pain. Infertility was determined among women reporting no birth control or methods considered being unreliable, including withdrawal, rhythm method, vasectomy, or using the following methods rarely or occasionally:diaphragm, condoms, spermicidal foam/cream/jelly/suppositories, or cervical cap. Infertility was defined by lack of conception despite unprotected intercourse during 12 or more months of followup. Self-reported pregnancy was determined by a positive urine/blood test or physician's diagnosis among all women in the cohort. Live birth was determined among all women in the cohort by self-report during followup. Recurrent PID was self-reported and verified whenever medical records were available (45% of cohort). Women were considered to have recurrent PID if they experienced a subsequent episode of PID more than 30 days after the index illness. Chronic pelvic pain was defined by two or more consecutive reports of pelvic pain during telephone followup interviews administered through 32 months [14]. This translates to approximately 6 months or greater duration of pain [14]. Data from at least 2 followup visits were required to determine chronic pelvic pain. 

Pmp antibody levels were previously measured among a subset of 40 *C. trachomatis*-positive women who had stored serum samples available. Pmp antibody levels were measured in densitometric units. These methods have been described elsewhere [5]. Briefly, purified EBs or partially purified rPmp polypeptides (rPmpD-FL and rPmpI-N) were subjected to sodium dodecylsulfate-polyacrylamide gel electrophoresis (SDS-PAGE). Amounts of rPmps were standardized by immunoblot analysis with anti-His tag antibody (1 : 1,500; GE Healthcare). Blots were visualized using Molecular Dynamics Typhoon 9400 imager (Amersham Biosciences, NJ). Antibody reactivity against the highest molecular-mass band in each lane was analyzed using Image Quant 5.2 image analysis software (Molecular Dynamics Sunnyvale, Calif). Serum response against each rPmp was quantified by the volume of the band. Data were normalized against His-tag-specific antibody reactivity. 

Since PmpA, PmpD, and PmpI are uniquely expressed and may play a role in chlamydial pathogenesis [9, 10], we chose to only include these Pmps in our analyses. Our objective was to determine if markers of inflammation or sequelae differed between women who displayed antibody reactivity to PmpA, PmpD, or PmpI and those who did not. Both continuous and binary variables were used. Differences in baseline characteristics were compared between groups using Chi-square or Fisher's exact tests and *t*-test for the normally distributed continuous variables. Chi-square or Fisher's exact tests were also used to compare differences in the frequency of inflammatory markers (elevated white blood cell count (WBC), temperature, elevated C-reactive protein (CRP), bilateral adnexal tenderness, cervicitis, endometritis, upper genital tract infection (UGTI), erythrocyte sedimentation rate (ESR)), and reproductive outcomes (infertility, pregnancy, live birth, chronic pelvic pain, and recurrent PID) between groups. We also examined these relationships using a continuous variable for Pmp antibody response. As the distributions of PmpA, PmpD, and PmpI were skewed, nonparametric tests were used. 

We also sought to determine if high levels of Pmp antibody expression were associated with inflammation or sequelae. After running sensitivity analyses, antibody reactivity groups for PmpD and PmpI were defined using median cut-points (high reactivity: PmpD ≥ 0.41; PmpI ≥ 0.89 and low reactivity: PmpD < 0.41; PmpI < 0.89). Due to the small number of women who expressed antibodies to PmpA, we were unable to examine levels of antibody expression (*n* = 5). Logistic regression was used to calculate odds ratios (OR) and 95% confidence intervals (CI). Cox regression was used to calculate hazard ratios and 95% CI for time-to-pregnancy and time-to-recurrent PID. Models were adjusted for age and race. Additionally, time to pregnancy was adjusted for history of infertility, which was self-reported at baseline. If any model had less than 5 in any cell, it was excluded from regression analysis. All analyses were completed using SAS V9.2 (Cary, NC).

## 3. Results

Overall, women in this cohort tended to be less than 25 years of age (85.0%), African American (77.5%), single (86.8%), and having at least a high school education (60.0%). At baseline, the majority of women reported abnormal vaginal discharge (62.5%), had bilateral adnexal tenderness (80.0%) and mucopurulent cervicitis (65.7%), and had chlamydia isolated from the cervix only (58.3%). Women who expressed antibody to PmpI were more likely to smoke compared to women who did not express PmpI antibody (56.7% versus 20.0%; *P* = 0.0411) (Table 1). There were no other significant differences in important baseline characteristics between women who expressed antibody to PmpA, PmpD, or PmpI and women who did not. 

Results show that compared to women who did not express antibody to PmpA, rates of elevated WBC (40.0% versus 23.5%), increased CRP (66.7% versus 46.2%), increased ESR (40.0% versus 31.4%), endometritis (100% versus 60.7%), and UGTI (75.0% versus 46.8%) were higher among women who expressed antibody to PmpA (Table 2). However, these differences did not reach statistical significance. Similarly, there were no significant differences in the frequency of infertility, recurrent PID, or chronic pelvic pain between groups. However, only 40% of women with antibody reactivity to PmpA achieved pregnancy compared to 85.7% of women who did not express antibody reactivity to PmpA (*P* = 0.042). In addition, none of the women with antibody reactivity to PmpA had a live birth, while 80% of women without antibody reactivity to PmpA had a live birth (*P* = 0.005). When examined as a continuous variable the results did not differ. Expression of anti-PmpA antibody was significantly increased in women who did not achieve pregnancy (*P* = 0.0192) or did not achieve a live birth (*P* = 0.0043).

There were no significant differences in inflammatory markers or reproductive sequelae between women who displayed antibody reactivity to PmpD and women who did not (Table 3). Results did not change when an antibody response to PmpD was considered as a continuous variable. Results were similar for PmpI (Table 4). However, women expressing antibody to PmpI were more likely to have UGTI compared to women who did not express antibody to PmpI (61.5% versus 20.0%; *P* = 0.026). Women who expressed antibody to PmpI were slightly more likely to have endometritis (72.7% versus 50.0%) although this did not reach statistical significance (*P* = 0.2096). When PmpI antibody response was examined as a continuous variable, the mean expression did not significantly differ between those with UGTI and those without UGTI (*P* = 0.276).

When levels of antibody expression for PmpD and PmpI were examined, there were no significant differences in the frequency of inflammatory markers or reproductive sequelae between high and low antibody reactivity groups. Although nonsignificant, women with high PmpD reactivity were slightly more likely to have an elevated WBC count (33.3% versus 16.7%), elevated ESR (42.9% versus 21.1%), elevated CRP (62.5% versus 37.5%), mucopurulent cervicitis (77.8% versus 52.9%), and endometritis (75.0% versus 56.3%) compared to women with low PmpD reactivity. Women with high PmpI reactivity were slightly more likely to have elevated CRP (55.6% versus 42.9%), bilateral adnexal tenderness (85.0% versus 75.0%), mucopurulent cervicitis (72.2% versus 58.8%), endometritis (71.4% versus 61.1%), and UGTI (58.8% versus 42.1%) compared to women with low PmpI reactivity although this did not reach statistical significance. Logistic regression also revealed no significant associations. 

Similarly, a nonsignificant decrease in pregnancy rates (adjusted hazard ratio (AHR) 0.7, 95% CI 0.3–1.6) and increase in recurrent PID were observed for women with high PmpD antibody reactivity (AHR 1.3, 95% CI 0.2–8.3) (Table 5). In contrast, high PmpI antibody reactivity had minimal effects on sequelae. However, after adjustments, women with high PmpI antibody reactivity showed a nonsignificant trend towards decreased live births (AOR 0.6, 95% CI 0.1–4.0).

## 4. Discussion

Among women with mild-to-moderate chlamydial PID, those who expressed antibody to PmpA were less likely to achieve pregnancy and less likely to report a live birth. The overall effects of seropositivity for PmpD and PmpI on inflammation and reproductive sequelae were minimal. However, women who expressed PmpI antibody were more likely to have UGTI. Trends towards elevated baseline genital tract and systemic inflammation, increased reproductive morbidity, and decreased pregnancy rates were observed among women with high PmpD antibody reactivity. Similarly, women with high PmpI antibody reactivity displayed trends towards elevated baseline inflammation. However, these results were nonsignificant. 

The progression to PID following lower genital *C. trachomatis * infection varies. Among high-risk groups, 2–4.5% of women with untreated chlamydial infection will develop PID within 2 weeks, and 19% with treated chlamydial infection will develop PID within 3 years [1, 15–18]. Chlamydial virulence proteins may explain differences in PID progression. As PmpA, PmpD, and PmpI have relatively low “off” frequencies (0.5–1%) [9], they should be present for antigenic processing and presentation allowing for antibody induction in the majority of infected individuals. In fact, PmpD and PmpI antibodies were frequent in our cohort. However, the prevalence of PmpA-expressing inclusions in *in vitro*-grown *C. trachomatis* does not correlate with the the low frequency of PmpA antibodies detected in our cohort of women with clinical PID and in other populations of *C. trachomatis*-infected patients [5]. The reason for this discrepancy is not entirely clear. Tan et al. suggest that Pmp expression in *in vitro*-grown *C. trachomatis* differs from *C. trachomatis* in the human genital tract [9]. Chlamydiae processing and secretion of Pmp fragments may also differ, possibly resulting in varied antibody expression. It is also possible that PmpA may have poor immunogenicity or that PmpA antibodies were generated at low levels not recognized by the initial SDS-PAGE analysis. Still, PmpA, PmpD, and PmpI are the most conserved Pmps of *C. trachomatis*, and their expression is unaltered in response to stress [10]. This may suggest that PmpA, PmpD, and PmpI are important for chlamydial survival and may play a role in chlamydial pathogenesis [9, 10].

Not all women with PID go on to develop reproductive morbidity. Studies have found a link between tubal occlusion and chlamydial antibodies [1, 19], as well as chlamydial infection and post-PID infertility [1, 20]. Inflammation caused by chlamydial infection may play a role in the development of infertility, through damage to the cilia lining of the Fallopian tubes, Fallopian tube blockage or closure, or adhesion formation among pelvic organs [2]. Our data indicate that women who express PmpA antibody were less likely to achieve pregnancy and less likely to have a live birth. Pregnancy and live birth can be used as markers of fertility. These variables are easier to define compared to infertility which requires classification of contraception than can be hampered by changing variables over time, concurrent use of more than 1 method of contraception, missing data, and compliance. Therefore, our results may suggest that PmpA plays a role in upper genital tract pathology. Although rates of inflammatory markers were increased among women who express PmpA antibody, no statistically significant differences were found between the groups. These null findings could be a result of our limited power. Due to the low frequency of PmpA in our cohort, we were also unable to examine levels of antibody titer. 

Data suggest that PmpD acts as an adhesion molecule and stimulates proinflammatory cytokines through the nuclear factor-*κ*B pathway [7, 8]. Since PmpD may stimulate host cell inflammatory responses, it is possible that increased antibody to PmpD reflects increased exposure to these potentially pathogenic ligands. We did find increased inflammation and reproductive sequelae among women with high antibody titers to PmpD. However, these results were nonsignificant. Overall, expression of PmpD antibody appeared to have minimal effects on inflammation and reproductive sequelae in this study. Crane et al. reported that anti-PmpD antibodies result in neutralization of chlamydial elementary bodies and reduced infectivity *in vitro* [6]. In our *in vivo* study, we found no evidence for protection from disease in women with high seropositivity to PmpD. However, it should be noted that as our study used recombinant denatured material for the measurement of seroreactivity, *in vivo *antibody reactivity to native PmpD present on chlamydial elementary bodies may not be fully reflected.

Tan et al. found that the PEACH PID population had significantly higher levels of PmpI antibodies compared to adolescent females with lower genital tract infection (*P* < 0.0001) [5]. This could suggest that high titers to PmpI could be associated with chlamydial progression to the upper genital tract [10]. We did find that women with antibody reactivity to PmpI were more likely to have UGTI. Endometritis was also more frequent in this group although results were nonsignificant. When we examined PmpI as a continuous variable, we did not find a significant difference in PmpI antibody expression between women with UGTI and women without UGTI. Therefore, this finding may have been due to chance. Women from the PEACH cohort are older and have likely been exposed to *C. trachomatis* more often than adolescents. Screening studies have found that older women have less infection but increased chlamydial antibodies compared to adolescents [3]. Therefore, high antibody reactivity to Pmps could represent a measure of cumulative chlamydial exposure, indicating that women with more infections suffer greater sequelae. In fact, a study among 443 PEACH participants found that PID recurrence was higher (HR 2.48, 95% CI 1.00–6.27), and pregnancy rates were significantly lower (HR 0.47, 95% CI 0.28–0.79) among women whose antibody titers to chlamydia EB were in the highest tertile [21]. However, we were unable to find any significant associations between high antibody reactivity to PmpI or PmpD and reproductive sequelae. Further, we found no significant associations with markers of inflammation. 

There could be several reasons for our mostly null findings. All women in our cohort had clinically suspected PID. Therefore, we were unable to compare women with chlamydial PID to women with uncomplicated *C. trachomatis* infection. Future studies should compare these groups to determine the role of Pmps in *C. trachomatis* progression. In addition, our sample size limited our power to detect significant associations. We must also consider other factors that may play a role in chlamydial pathogenesis. It is possible that host susceptibility may explain why some women with chlamydia experience sequelae and others do not. In fact, chlamydia is suggested to be a disease of immunopathology [3]. Therefore, genetic variations in host immune receptors may cause unfavorable inflammation and explain the variability in outcomes. Chlamydial load may also play a role in the course and outcome of infection. We did find a borderline association with cervicitis among women with high PmpD reactivity using logistic regression, and chlamydial cervicitis has been associated with a higher chlamydial load [22]. 

To our knowledge, this is the first study to examine the role of Pmp antibody response in inflammation and post-PID sequelae. Data were obtained from a prospective randomized clinical trial with comprehensive demographic, clinical, and obstetric measurements. Further, our findings are generalizable to patients treated for clinically suspected PID. However, as some patients with clinically suspected PID might actually have ovarian cysts, pelvic adhesions, or endometriosis [23], some women in our study may not have had true upper genital tract infection. We attempted to minimize some of this misclassification by excluding women reporting greater than 30 days of pain at the time of enrollment. We recognize that reproductive outcomes were based on self-reported data and that misclassification bias is possible. Our analysis of Pmp seropositivity is semiquantitative and cannot generate actual titers. However, it is still sufficient for comparative rough estimates of Pmp-subtype-specific titers. In addition, antibody expression may differ depending on the time course of the infection. We are unable to confirm when women first became infected with *C. trachomatis*. All women were recruited when they presented for care for symptoms. We do know that time to treatment does differ among women with PID [24]. Time to treatment did not significantly differ between any of our groups. Serovars for *C. trachomatis* were not determined in the PEACH study. However, PmpA, PmpD, and PmpI are the most conserved Pmps of *C. trachomatis*, and it is possible that their functions are also conserved across serovars [9, 10]. As this pilot study is limited by power, larger studies should continue to explore the role of Pmps in the course and outcome of *C. trachomatis* infection. Specifically, the correlation between PmpA antibody reactivity and reproductive sequelae needs to be confirmed.

Variability in the progression of chlamydial infection may be due to varied expression of chlamydial Pmps that are reflected by the serum anti-Pmp antibody response. Our data suggest that women who express PmpA antibody had decreased pregnancy rates and decreased live births. Rates of inflammatory markers were increased among women with PmpA antibody although these results were nonsignificant. In contrast, a positive antibody response to PmpD or PmpI appeared to relate minimally to reproductive sequelae and inflammation. Results were the same when high antibody reactivity to both PmpD and PmpI was explored. These results suggest a possible role for PmpA, but not for PmpD or PmpI, in upper genital tract pathology. 

## Figures and Tables

**Table 1:** Baseline characteristics of women by Pmp antibody expression.

Characteristics	PmpA			PmpD			PmpI		
	No *n* = 35	Yes *n* = 5	*P* value	No *n* = 10	Yes *n* = 30	*P* value	No *n* = 10	Yes *n* = 30	*P* value
Demographics									

Age									
<25 years	30 (85.7)	4 (80.0)	0.7378	8 (80.0)	26 (86.7)	0.3213	8 (80.0)	26 (86.7)	0.3213
Race/ethnicity African American	27 (77.1)	4 (80.0)	0.8862	8 (80.0)	26 (76.7)	0.3350	8 (80.0)	23 (76.7)	0.3350
Married	4 (12.1)	1 (20.0)	0.6272	1 (10.0)	4 (14.3)	0.4079	1 (10.0)	4 (14.3)	0.4079
Uninsured	11 (33.3)	3 (60.0)	0.0876	6 (60.0)	18 (64.3)	0.2850	6 (60.0)	18 (64.3)	0.2850
Education less than high school	14 (40.0)	2 (40.0)	1.000	2 (20.0)	14 (87.5)	0.1091	2 (20.0)	14 (46.7)	0.1091

Clinical									

*Neisseria gonorrhoeae*	8 (25.8)	2 (50.0)	0.3134	3 (37.5)	7 (25.9)	0.2709	2 (22.2)	8(30.8)	0.3064
*Mycoplasma genitalium*	3 (12.5)	1 (25.0)	0.3954	1 (25.0)	3 (12.5)	0.3954	1 (16.7)	3 (13.6)	0.4513
Bacterial vaginosis	21 (67.7)	1 (20.0)	0.0584	5 (55.6)	17 (62.9)	0.2800	5 (55.6)	17 (63.0)	0.2800
History of PID	10 (28.6)	1 (20.0)	0.3970	4 (40.0)	7 (23.3)	0.1849	3 (30.0)	8 (26.7)	0.3038
History of chlamydia	15 (45.5)	3 (60.0)	0.3089	4 (44.4)	14 (48.3)	0.7183	5 (55.6)	13 (44.8)	0.4272
^ a^Pelvic pain (mean score ± SD)	65 ± 22	75 ± 15	0.3605	69 ± 14	66 ± 23	0.7387	65 ± 16	67 ± 17	0.7810
^ b^Days of pain(mean score ± SD)	8 ± 7	5 ± 2	0.3238	6 ± 6	9 ± 7	0.3637	7 ± 7	8 ± 7	0.7569

Behavior									

Current smoker	15 (42.9)	4 (80.0)	0.1237	4 (40.0)	5 (50.0)	0.2481	2 (20.0)	17 (56.7)	0.0411
Drug use	12 (34.3)	2 (40.0)	0.3596	4 (40.0)	10 (33.3)	0.2719	4 (40.0)	10 (33.3)	0.2719

^a^( mean of current pelvic pain score, average pelvic pain score and worst pelvic pain score) × 10; ^b^Self-reported time to treatment following onset of symptoms.

**Table 2:** Frequency of baseline inflammatory markers and reproductive sequelae by PmpA antibody expression.

Inflammation and sequelae	PmpA		
	No *n* = 35	Yes *n* = 5	*P* value
Inflammation			

^ a^Elevated temperature (>100.4°F)	0 (0.0)	1 (25.0)	0.1111
^ a^Elevated WBC count (>10,000 mm^3^)	8 (23.5)	2 (40.0)	0.2856
Erythrocyte sedimentation rate (>15 mm/hr)	11 (31.4)	2 (40.0)	0.7019
^ a^C-reactive protein (>5 mg/dL)	6 (46.2)	2 (66.7)	0.5218
Bilateral adnexal tenderness	28 (80.0)	4 (80.0)	1.000
^ a^Mucopurulent cervicitis	20 (66.7)	3 (60.0)	0.7712
Upper genital tract infection	15 (46.7)	3 (75.0)	0.2494
^ a^Endometritis	17 (60.7)	4 (100.0)	0.1664

Reproductive sequelae			

Infertility	5 (14.3)	0 (0.0)	0.3663
^ b^Live birth	20 (80.0)	0 (0.0)	0.0053
Pregnancy	30 (85.7)	1 (40.0)	0.0422
^ b^Chronic pelvic pain	13 (38.2)	0 (0.0)	0.1142
^ b^Recurrent PID	6 (17.7)	0 (0.0)	0.4122

^a^WBC data was available for 39 patients, CRP data was available for 16 patients, mucopurulent cervicitis data was available for 35 patients, UGTI data was available for 36 patients, and endometritis data was available for 32 patients; ^b^live birth data was available for 29 patients; chronic pelvic pain and recurrent PID data were available for 39 patients.

**Table 3:** Frequency of baseline inflammatory markers and reproductive sequelae by PmpD antibody expression.

Inflammation and sequelae	PmpD		
	No *n* = 10	Yes *n* = 30	*P* value
Inflammation			

^ a^Elevated temperature (>100.4°F)	0 (0.0)	1 (3.7)	0.7500
^ a^Elevated WBC count (>10,000 mm^3^)	2 (20.0)	8 (27.6)	0.3038
Erythrocyte sedimentation rate (>15 mm/hr)	3 (30.0)	10 (33.3)	0.2996
^ a^C-reactive protein (>5 mg/dL)	2 (40.0)	6 (54.6)	0.3590
Bilateral adnexal tenderness	7 (70.0)	25 (83.3)	0.2224
^ a^Mucopurulent cervicitis	4 (50.0)	19 (70.4)	0.1862
Upper genital tract infection	4 (50.0)	17 (70.8)	0.1878
^ a^Endometritis	3 (33.3)	15 (55.6)	0.1609

Reproductive sequelae			

Infertility	1 (10.0)	4 (13.3)	0.4165
^ b^Live birth	7 (77.8)	13 (65.0)	0.2787
Pregnancy	8 (80.0)	24 (80.0)	1.000
^ b^Chronic pelvic pain	4 (44.4)	9 (30.0)	0.2219
^ b^Recurrent PID	2 (22.2)	4 (13.3)	0.3024

^a^WBC data was available for 39 patients, CRP data was available for 16 patients, mucopurulent cervicitis data was available for 35 patients, UGTI data was available for 36 patients, and endometritis data was available for 32 patients; ^b^ live birth data was available for 29 patients; chronic pelvic pain and recurrent PID data were available for 39 patients.

**Table 4:** Frequency of baseline inflammatory markers and reproductive sequelae by PmpI antibody expression.

Inflammation and sequelae	PmpI		
	No *n* = 10	Yes *n* = 30	*P* value
Inflammation			

^ a^Elevated temperature (>100.4°F)	0 (0.0)	1 (3.7)	0.7500
^ a^Elevated WBC count (>10,000 mm^3^)	2 (20.0)	8 (27.6)	0.3038
Erythrocyte sedimentation rate (>15 mm/hr)	3 (30.0)	10 (33.3)	0.2996
^ a^C-reactive protein (>5 mg/dL)	2 (66.7)	6 (46.2)	0.4000
Bilateral adnexal tenderness	6 (60.0)	26 (86.7)	0.0748
^ a^Mucopurulent cervicitis	5 (62.5)	18 (66.7)	0.3145
Upper genital tract infection	2 (20.0)	16 (61.5)	0.0263
^ a^Endometritis	5 (50.0)	16 (72.7)	0.1457

Reproductive sequelae			

Infertility	1 (10.0)	4 (13.3)	0.4165
^ b^Live birth	6 (75.0)	14 (66.7)	0.3251
Pregnancy	8 (80.0)	24 (80.0)	1.000
^ b^Chronic pelvic pain	2 (20.0)	11 (37.9)	0.2996
^ b^Recurrent PID	3 (30.0)	3 (10.3)	0.1344

^a^WBC data was available for 39 patients, CRP data was available for 16 patients, mucopurulent cervicitis data was available for 35 patients, UGTI data was available for 36 patients, and endometritis data was available for 32 patients; ^b^ live birth data was available for 29 patients; chronic pelvic pain and recurrent PID data were available for 39 patients.

**Table 5:** Effect of Pmp antibody reactivity on time-to-pregnancy and time-to-recurrent PID.

Subgroup	Pregnancy		Recurrent PID	
	Crude HR (95% CI)	^ a^Adjusted HR (95% CI)	Crude HR (95% CI)	^ a^Adjusted HR (95% CI)
^ b^ *High PmpD *(*n* = 28)	0.7 (0.3–1.3)	0.7 (0.3–1.6)	0.9 (0.2–4.7)	1.3 (0.2–8.3)
^ c^ *High PmpI *(*n* = 23)	1.3 (0.7–2.7)	1.4 (0.7–3.0)	0.6 (0.1–3.0)	0.7 (0.1–5.3)

^a^Models were adjusted for age, race, and models predicting infertility, pregnancy, and live birth were additionally adjusted for infertility self-reported at baseline; ^b^PmpD antibody reactivity is based on a median cut-point; low reactivity <0.41, high reactivity ≥0.41; ^c^PmpI antibody reactivity is based on a median cut-point; low reactivity <0.89, high reactivity ≥0.89.
